# Novel Transcription Factor Variants through RNA-Sequencing: The Importance of Being “Alternative”

**DOI:** 10.3390/ijms16011755

**Published:** 2015-01-13

**Authors:** Margherita Scarpato, Antonio Federico, Alfredo Ciccodicola, Valerio Costa

**Affiliations:** 1 Institute of Genetics and Biophysics “Adriano Buzzati-Traverso”, National Research Council, 80131 Naples, Italy; E-Mails: margherita.scarpato@igb.cnr.it (M.S.); antonio.federico@igb.cnr.it (A.F.); alfredo.ciccodicola@igb.cnr.it (A.C.); 2 Department of Science and Technology, University of Naples “Parthenope”, 80143 Naples, Italy

**Keywords:** alternative splicing, RNA-sequencing, transcription factors, ZNF266, KRAB (Krüppel-associated box) domain

## Abstract

Alternative splicing is a pervasive mechanism of RNA maturation in higher eukaryotes, which increases proteomic diversity and biological complexity. It has a key regulatory role in several physiological and pathological states. The diffusion of Next Generation Sequencing, particularly of RNA-Sequencing, has exponentially empowered the identification of novel transcripts revealing that more than 95% of human genes undergo alternative splicing. The highest rate of alternative splicing occurs in transcription factors encoding genes, mostly in Krüppel-associated box domains of zinc finger proteins. Since these molecules are responsible for gene expression, alternative splicing is a crucial mechanism to “*regulate the regulators*”. Indeed, different transcription factors isoforms may have different or even opposite functions. In this work, through a targeted re-analysis of our previously published RNA-Sequencing datasets, we identified nine novel transcripts in seven transcription factors genes. *In silico* analysis, combined with RT-PCR, cloning and Sanger sequencing, allowed us to experimentally validate these new variants. Through computational approaches we also predicted their novel structural and functional properties. Our findings indicate that alternative splicing is a major determinant of transcription factor diversity, confirming that accurate analysis of RNA-Sequencing data can reliably lead to the identification of novel transcripts, with potentially new functions.

## 1. Introduction

Human genome sequencing and large-scale international projects have highlighted that eukaryotic complexity does not correlate with genome size and gene number [1,2,3]. Further advances in sequencing technologies have revealed that more than 95% of human genes undergo alternative splicing (AS) [4]. This process leads to the production of multiple transcripts from a single gene, explaining the discrepancy between the low number of genes and the proteomic diversity [5,6]. Alternative splicing is a crucial regulatory mechanism in stem cell renewal and differentiation, organ morphogenesis, immune system specification and neural development. In some cases, alternative splicing has been demonstrated to have a causal role in disease onset [7]. Transcripts generated by AS have different spatial and/or temporal expression [8]. Exon skipping is the most prevalent AS mechanism in higher eukaryotes and reaches 40% of total AS in human [7,9].

Genes encoding transcription factors (TFs)—in human and mouse genomes—have the highest rate of AS [10]. This mechanism has been proposed to facilitate tissue- or cell-specific gene expression regulation, particularly during development [11]. Indeed, in many cases, the generation, through AS, of different TF isoforms can determine distinct, or even opposite, functions. It can be partially explained by the modular composition of TFs and by the recurrence of AS in modular exons that encode part of functional protein domains. AS can occur in DNA-binding, transactivation or dimerization domains of TFs. The resulting alternative isoforms can be translated into proteins with altered (increased or decreased) functionality, or these may display dominant negative activity [12,13]. The KRAB (Krüppel-associated box) is one of the domains most affected by AS in humans [10]. It is located at the amino-terminal region of the majority of Cys_2_His_2_ zinc finger (ZNF) proteins and is responsible for transcriptional repression through binding to corepressor proteins [14]. In this scenario, AS represents a previously underestimated mechanism to provide new structural/functional TF variants.

In recent years, the diffusion of next generation sequencing (NGS) technologies—particularly RNA-Sequencing (RNA-Seq)—has exponentially increased our ability to identify new transcripts generated by AS. Recent studies based on RNA-Seq have indicated that AS can affect cell phenotype and cause disease [15]. Nonetheless, its contribution to disease onset and progression is still unexplored. Thus, using RNA-Seq datasets [16], we recently developed a “transcript-centric” web resource focused on chromosome 21 (HSA21) genes, designed to study at the isoform level, rather than at gene level, Down syndrome and other HSA21-related genetic diseases [17]. However, despite that large-scale studies from research groups and/or international consortia [1,2] have expanded the landscape of AS in humans, most predicted isoforms still remain to be experimentally confirmed and characterized. This challenge is particularly relevant for genes encoding TFs, given the higher rates of AS in this group compared to other human genes.

Thus, starting from the re-analysis of our previously published RNA-Seq datasets [16], here we describe the identification and experimental validation of novel transcripts of seven TFs encoding genes generated by differential exon usage.

## 2. Results

### 2.1. In Silico Identification of New Splice Isoforms of Genes Encoding Transcription Factors (TFs)

**Figure 1:**
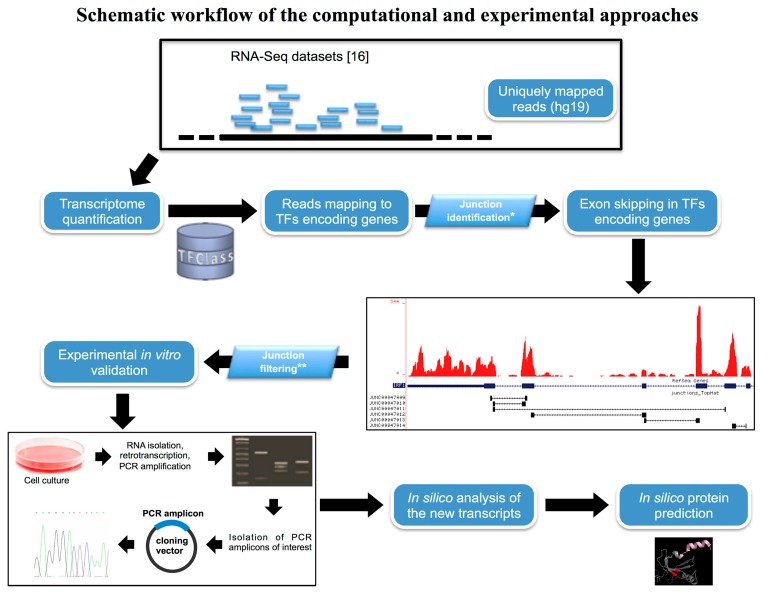
Computational and experimental workflow. Schematic overview of the *in silico* procedures used to infer the presence of new transcription factors (TFs) transcripts from the re-analysis of our RNA-Seq datasets. The experimental approach used to validate the presence of the new variants is also depicted.

We used our published RNA-Seq data [16] to identify new transcripts that encode transcription factors (see workflow scheme in Figure 1). First, we found that 1043 (out of ~1500) genes annotated in the Transcription Factor Class (TFClass) database [18] are expressed according to our RNA-Seq data. Normalized gene expression data revealed that most of genes encoding TFs have medium to low expression (Figure S1). Targeted re-analysis (Section 4.1 and [17]) of AS in genes encoding TFs revealed the presence of new putative isoforms, mainly originated by cassette exon skipping. A complete—or even partial—overlap with transcripts annotated in AceView [19] and expressed sequence tags (ESTs; [20]) databases was used as the selection parameter. In contrast, splice junctions with less than two mapped reads/sample and those that overlap genomic repeats were not considered for further studies. The 50 bp single-end libraries of our previous experiment did not allow us to automatically reconstruct the entire exon/intron structure of the new transcripts, nor to measure their relative abundance [16]. Thus, we *in silico* assessed the presence of new transcripts through a targeted analysis of “uniquely mapped” reads spanning along different exons. Fourteen new potential AS events in twelve genes encoding TFs were identified (Figure S1). We experimentally confirmed nine transcripts, whereas five could not be *in vitro* validated and were not considered in the further analyses.

**Figure 2:**
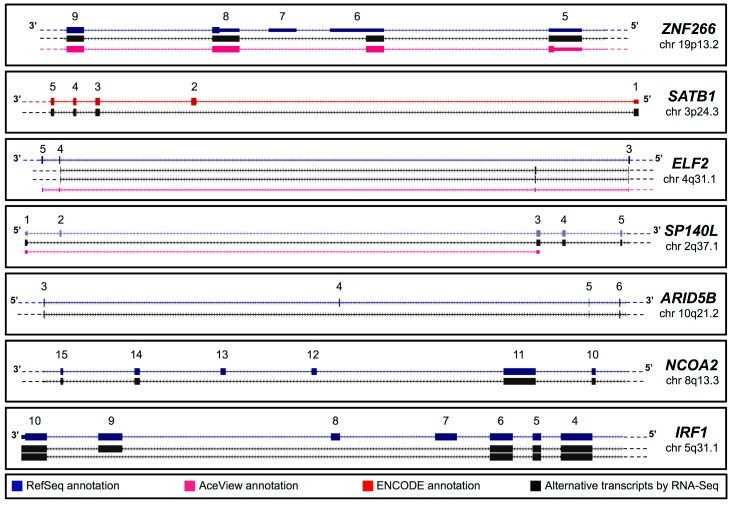
Schematic representation of newly identified TF transcripts. Newly identified transcripts encoding TFs (black)—*ZNF266*, *SATB1*, *ELF2*, *SP140L*, *ARID5B*, *NCOA2* and *IRF1*—are schematically compared to known gene annotations: RefSeq (blue), AceView predictions (purple) and Gencode (red).

A schematic summary of the validated transcripts *ZNF266*, *SATB1*, *ELF2*, *SP140L*, *ARID5B*, *NCOA2* and *IRF1* genes is shown in Figure 2. These AS events were classified in (1) “exon skipping” and (2) “exon gain” with alternative AUG usage; (3) “lack of modular exons” with open reading frame (ORF) maintenance and (4) “exon skipping with frameshift and premature termination codon (PTC) formation”. As schematized in Figure 2, the new *ZNF266* belongs to the first category, as it lacks part of the exon 6 and the entire exon 7 (RefSeq NM_006631.3 and NM_001271314.1). Similarly, the novel splicing event described for the *SATB1* gene consists of the skipping of exon 2 (GENCODE ENST00000440737.1), which creates a potential new AUG. RNA-Seq re-analysis also indicated the presence of two new transcripts of *ELF2* gene, originated by exon gain within intron 3. This event determines the usage of alternative AUG (RefSeq NM_201999.2; details are given in Figure 2).

For *SP140L* and *ARID5B* genes, RNA-Seq data indicated the presence of transcripts that lack exon 2 and exon 4, respectively (NM_138402.4 of *SP140L* and NM_032199.2 of *ARID5B*).

Interestingly, we also detected a new AS event (skipping of exons 12–13) for the *NCOA2* gene (RefSeq NM_006540.2). It generates a transcript with a frameshift and PTC formation that is predicted to undergo nonsense-mediated decay (NMD) or to be translated into a truncated protein [21]. Finally, we detected two transcripts of the *IRF1* gene, still not annotated. One transcript, that skips exons from 7 to 9, still retains the ORF, the other has a frameshift with PTC formation due to the skipping of exons 7 and 8. Notably, although Lee and colleagues [22] have already described these isoforms, our pipeline correctly labeled them as “new” since their sequences are not annotated in any public database. However, we submitted these sequences to public genomic repositories (accession numbers. in Table 1) and we did not further consider these transcripts as “new”.

**Table 1:** Novel identified TFs transcripts with accession numbers and primer pairs used for RT-PCR validation.

Gene Symbol	Chromosome Position	Accession Number	Primer Sequence (5'–3')	
			Forward Primer	Reverse Primer
***ZNF266***	19p13.2	LN607832	GAAGTAGAAAGGGTGGTGGC	TTCTTGTAGTTCTCCAGCATC
***SATB1***	3p24.3	LN626687	CGTATGGGGAAAGAGGACAA	GCGTTTTCATAATGTTCCACC
***ELF2***	4q31.1	LN626691	GAGACCGAGAATGTGGAAAC	TACTGCTGTGAACTGATGCT
***ELF2***	4q31.1	LN626692	GAGACCGAGAATGTGGAAAC	TACTGCTGTGAACTGATGCT
***SP140L***	2q37.1	KF419365/6/7 *	GGTGGGACGATGGCAGGT	CAAGTCCCTCATCTACATCC
***ARID5B***	10q21.2	LN607831	AGGAATGGACAGAAGGAAGC	ATGGTTTCTTTTTGCGTGGTC
***NCOA2***	8q13.3	LN607830	GTGAGCCCCAAGAAGAAAGA	GACTCTCACAGCCGAACTC
***IRF1***	5q31.1	LN607829	CTCCACTCTGCCTGATGAC	GATGGAGGGCAACCGGACT
***IRF1***	5q31.1	LN626686	CTCCACTCTGCCTGATGAC	GATGGAGGGCAACCGGACT

* Previously submitted to GenBank database by Saare and colleagues.

### 2.2. Validation of Novel TFs Transcripts

RT-PCR was used to experimentally validate the new splicing events detected by RNA-Seq data re-analysis (see Experimental Section). Using the approach schematized in Figure 1, we confirmed by experimental evidence the presence of the above-discussed nine new transcripts. As previously mentioned, four out of 13 new AS events detected by RNA-Sequencing could not be experimentally confirmed, possibly due to their expression at levels much lower than the canonical variants. Table 1 lists *in vitro* validated TF transcripts. As the most relevant results were obtained for the *ZNF266* gene, all related results are discussed in a separate paragraph (Section 2.4).

**Figure 3:**
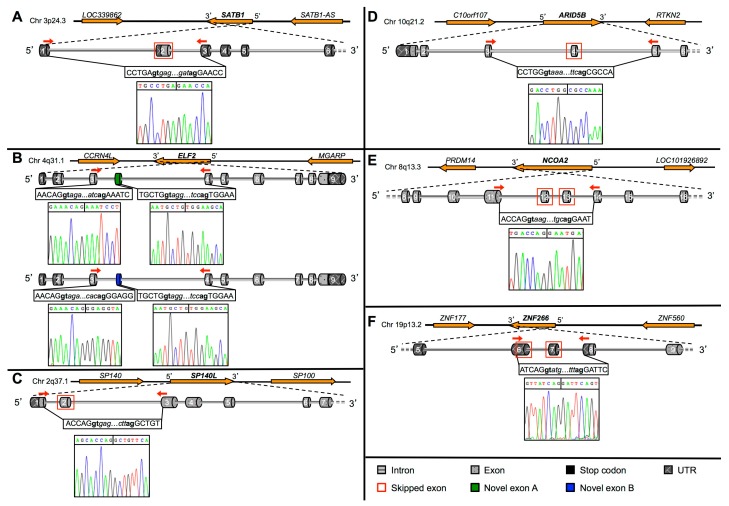
Graphical representation of newly identified splicing events in TF genes. General scheme of the new alternative splicing events identified for *SATB1* (**A**); *ELF2* (**B**); *SP140L* (**C**); *ARID5B* (**D**); *NCOA2* (**E**) and *ZNF266* (**F**). For all the genes, the genomic region encompassing the gene is shown in the upper part. Nucleotide sequences (and electropherograms by Sanger sequencing) of the new splice junctions are shown below the exon/intron structure for each gene. White numbers indicate exons’ numbers. Donor and acceptor splice sites are shown in bold. Red arrows indicate the primers annealing sites.

*SATB1* gene validation assay revealed a shorter amplicon of 245 bp with respect to the 480 bp annotated one (ENST00000440737.1). Sanger sequencing confirmed the skipping of exon 2 in this new transcript (Figure 3A). For the *ELF2* gene we found a more complex *scenario*. Indeed, we validated two new transcripts for this gene. Particularly, targeted PCR analysis revealed three amplicons (46, 149 and 205 bp). By cloning and sequencing we observed that only the shortest one (46 bp) corresponded to the annotated *ELF2* transcript (NM_201999.2). Indeed, the analysis of nucleotide sequences confirmed RNA-Seq data, *i.e.*, the presence of two novel exons (schematized in Figure 2 and Figure 3B). In detail, the longest amplicon (205 bp) corresponds to a new *ELF2* transcript containing an additional exon (159 bp long) located within intron 3 of the *ELF2* gene. Surprisingly, sequence analysis on the intermediate PCR amplicon (149 bp) revealed the presence of a novel transcript that originates by AS from the new above-described transcript, which has a cryptic acceptor splice site in the new exon. Therefore, this AS leads to the formation of another exon, 103 bp long. Notably, both these exons are reported in the AceView database, although these predictions refer to non-coding transcripts with 5' and 3' alternative exons with respect to the RefSeq transcript.

We also confirmed exon 2 skipping in the new transcript of* SP140L* gene, as indicated by a shorter (79 bp) PCR product, further confirmed by Sanger sequencing (Figure 3C). BLAST-Like Alignment Tool (BLAT) analysis revealed that this sequence overlaps a gene prediction reported in AceView (SP140LandSP100andHMGB1L3.wmAug10). The *bona fide* presence of this new transcript is also supported by three GenBank entries (KF419365, KF419366, KF419367) and two ESTs (DW441701 and DW428200) that overlap the splice junction. Similarly, the presence of a 136 bp product, shorter than the RefSeq annotated transcript (367 bp), confirmed exon 4 skipping in the *ARID5B* gene (Figure 3D). Sequence analysis revealed this new transcript overlaps two ESTs (BG536156 and CD704939).

The above described AS events determine the lack of modular exons with ORF maintenance. Differently, the exon skipping identified by RNA-Seq in *NCOA2* gene was predicted to determine PTC formation due to frameshift. A shorter amplicon (269 bp) in the RT-PCR analysis confirmed the presence of the new *NCOA2* transcript. Sequencing analysis confirmed the skipping of exons 12 and 13 (Figure 3E), revealing that the new transcript lacks 418 bp of the RefSeq annotated *NCOA2* transcript.

The *bona fide* presence of the new TFs transcripts, other than confirmed by experimental validations and ESTs entries is further supported by expression analysis on RNAs from cell lines and human tissues (Table S1 and Experimental Section). In addition, since we used our RNA-Seq data of early outgrowth endothelial circulating progenitor cells (eEPCs) from individuals with Down syndrome and euploid donors, we tested if new TFs variants are associated with the syndrome. Experimental validations carried out on a panel of trisomic and euploid RNAs from eEPCs used in previous studies [16,17,23] revealed that all the new TFs variants are expressed in both conditions.

### 2.3. Protein Prediction of the Novel TFs Isoforms

The expression of the new TFs transcripts has been confirmed *in vitro*. Nonetheless, we did not provide evidence of protein translation. Notably, for most of these transcripts, the presence of ORFs and the significant evolutionary conservation (Table S2) support the hypothesis of their translation. Indeed, ORF analysis revealed that the new transcripts of *SATB1*, *ELF2*, *SP140L* and* ARID5B* retain the ORF. Protein alignments are shown in Supplementary File S1. *SATB1* and *ELF2* are predicted to use different AUGs that are in an adequate Kozak context for the initiation of translation [24].

The novel* SATB1* transcript putatively creates a novel ORF, with an AUG, located 216 bp downstream from the canonical one. The new ORF is predicted to encode a shorter protein of 691 aa, that lacks the first 72 aa with respect to the 763 aa long annotated one (UniProt ID Q01826). This new putative isoform lacks the Nuclear Localization Signal (NLS; aa from 20 to 40). The 3D structure models of SATB1 proteins were predicted by homology modeling, and are shown in Figure S2.

Similarly, ORF analysis for the new *ELF2* transcripts revealed that exon gain determines the formation of a novel ORF (with a downstream AUG). Both transcripts encode a protein isoform of 477 aa, 104 shorter than the annotated ELF2 isoform 5 (Uniprot ID Q15723-5). The new protein—that corresponds to the predicted ETS-related (E26 transformation-specific-related) transcription factor Elf-2 isoform X4 (XP_005262863.1)—lacks the transactivation domain structured as a β-barrel as shown in the 3D prediction (Figure S3).

Unfortunately, for the new SP140L protein we could not predict any relevant effect since a few residues are lost (aa 12–36) in a non-structured region of the SP100 domain, whose function is still unknown.

The novel predicted ARID5B protein consists of 1111 amino acids, whereas the annotated one is 1188 aa long (UniProt ID Q14865). The putative new isoform lacks amino acids from 168 to 244. Since structure annotation in public databases was lacking for the ARID5B protein, we could not reconstruct an accurate 3D model for this protein. Nonetheless, we observed that the missing amino acids do not belong to any functional annotated domain. However, we cannot exclude that these missing amino acids affect structure, function and/or stability of the AT-rich interactive domain-containing protein 5B.

Finally, the novel exon-skipping event identified in the *NCOA2* gene determines frameshift with PTC formation. It is predicted to produce a putative isoform lacking 665 amino acids at the *C*-terminus (from the amino acid 799) with respect to the annotated protein (ID Q15596). Such protein would lack a LXXLL motif, a LLXXLXXXL motif and a Poly-Gln region. These regions mediate the NCOA2 heterodimerization. Similarly to the ARID5B protein, we could not reconstruct the 3D model of NCOA2 protein due to the lack of any structure annotation, useful as template, in public databases.

### 2.4. Identification and Characterization of a Novel ZNF266 Variant

There is much ambiguity about the *ZNF266* gene and protein in literature as well as in public databases. Indeed, according to the HUGO Gene Nomenclature Committee (HGNC), the gene *ZNF266*—that maps to 19p13.2—has one alias, i.e. *HZF1*. On the contrary, all PubMed indexed publications—and particularly the paper that describes for the first time the identification and characterization of *HZF1* [25]—use *HZF1* as synonym to indicate the *ZNF16* gene (a different gene that maps to 8q24.3). In this paper, we refer to the *ZNF266* gene (19p13.2).

**Figure 4:**
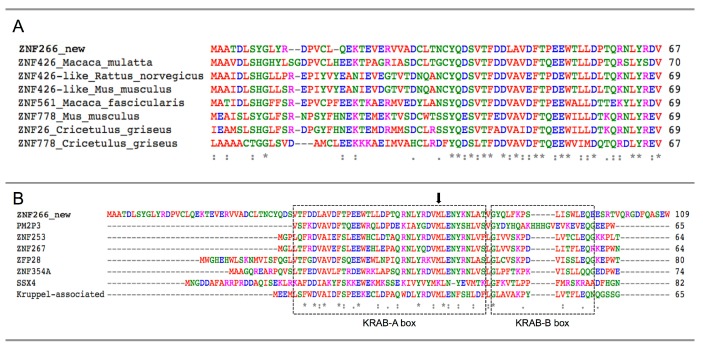
Multiple alignment of the *N*-terminal residues of the novel predicted ZNF266 variants. The evolutionary conservation of the new 67 amino acids is shown in (**A**); Krüppel-associated box (KRAB)-A and -B boxes (dashed lines) alignment with other human genes is shown in (**B**). Identical residues are indicated by “*”, conservation between groups of strongly and weakly similar properties by “:” and “.” respectively. Black arrow indicates the start of the canonical ZNF266 protein isoform.

**Figure 5:**
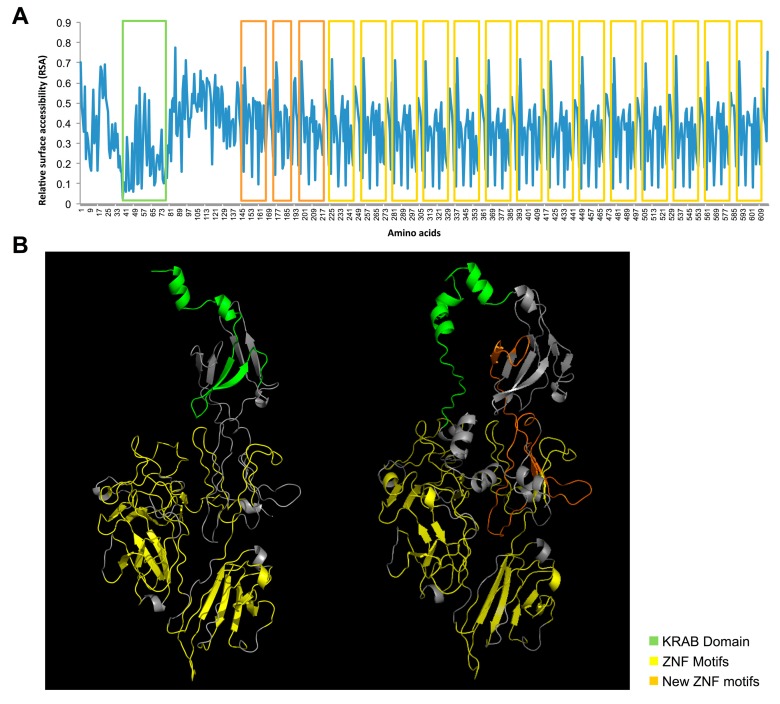
*In silico* characterization of ZNF266 predicted protein. Panel **A** shows relative surface accessibility (RSA) per residue; 3D structure of both annotated (**left**) and new (**right**) ZNF266 isoforms are shown in panel **B**. The protein backbone is shown in grey. The arrows show the direction of the beta-sheets, which is from the *N*- to the *C*-terminus. In both panels, functional domains and motifs are highlighted. In particular, the KRAB domain of the new ZNF266 isoform is shown in green, three predicted additional ZNF motifs in orange and the annotated ZNF motifs in yellow.

In detail, we amplified five PCR products (613, 458, 454, 324 and 158 bp; Figure S4A). Cloning and further sequencing revealed that four out of five amplicons corresponded to known *ZNF266* transcripts (uc010dwq.4, NM_006631.3, NM_001271314.1 and ENST00000592292.1). The shortest one confirmed the presence of the new transcript indicated by RNA-Seq, also supported by an AceView entry (ZNF266.bAug10; Figure 2 and Figure 3F). Sequence analysis of the new transcript revealed that the new AS event is predicted to cause an alternative AUG usage, in a strong Kozak context for the initiation of translation [24]. Indeed, 201 bp upstream the canonical AUG, we found a new putative translation-initiating site. Its usage would lead to the addition of 201 nucleotides to the ORF of the annotated *ZNF266*. The protein is predicted to have 67 new amino acids at *N*-terminus (Supplementary File S1). Local alignment of these 67 aa revealed a very high evolutionary conservation (Figure 4A). Diversity *per* residue calculation in multiple alignment showed that the amino acid diversity decreases in proximity of the canonical methionine (Figure S4B). The ZNF266 protein (UniProt ID Q14584) is 549 aa long, whereas the new isoform is 616 amino acids long. The annotated protein has a KRAB domain in position 1–42, corresponding to amino acids from 68 to 109 of the new protein. Interestingly, the analysis of conserved domains and comparative sequence analysis on the annotated ZNF266 protein revealed that the KRAB domain is incorrectly annotated in the UniProt database. Indeed, only the new predicted ZNF266 protein—with 67 additional amino acids at the *N*-terminus—has complete KRAB-A and KRAB-B box domains (amino acids from 39 to 77 and from 79 to 94, respectively; Figure 4B and Figure 5A). Moreover, the already annotated protein has 14 C_2_H_2_-type zinc finger motifs. Notably, solvent accessibility calculation per residue (Supplementary File S2) revealed a recurrence of maximum and minimum values in amino acids corresponding to these motifs. A similar occurrence was also present in three regions located in a hinge region (residues 88–100, 106–123 and 129–151 of the annotated protein, corresponding to amino acids 145–167, 173–190 and 196–218 of the new isoform). This observation suggested to us the presence of three additional zinc finger motifs located upstream of the already annotated ones (Figure 5A). In particular, amino acid alignments and solvent accessibility data revealed that predicted motifs are partially degenerated. *In silico* 3D structure of the new ZNF266 isoform predicted a structural rearrangement—due to the presence of 67 additional residues—in the region that mediates the transcriptional repression (Figure 5B). These evidences support the hypothesis that these additional residues may confer a functional role to the new ZNF266 variant herein identified.

## 3. Discussion

Alternative splicing is the most relevant molecular mechanism to expand the functional potentiality of a genome and increase proteome complexity, although its impact on cell physiology is still underestimated [7,9]. This is partially due to incomplete annotations, lack of experimental data and of transcript-specific expression profiles in cells/tissues and computational challenges. To partially fill the gap in AS knowledge, different web resources have been developed, such as ProSplicer [26], H-DBAS [27], whereas others have been recently dismissed or are currently not updated. In this regard, we recently developed an HSA21-centric database, ALE-HSA21, to provide transcript-based information and computational predictions for HSA21 genes [17]. Although it mainly focuses on Down syndrome and HSA21-related studies, it represents a first attempt to highlight the underestimated AS role in different research contexts. Large-scale transcriptome studies [1,2,3] have expanded the repertoire of human transcripts but most of these isoforms still remain to be experimentally confirmed and characterized.

Genes encoding transcription factors are among the most frequently spliced human genes [10]. A recent computational analysis of transcription-splicing integrated networks has shown that TFs are more extensively controlled by transcriptional regulation rather than by AS [28]. Nonetheless, the identification of different TF variants with a different—opposite and/or dominant negative—effect on target gene transcription supports the hypothesis that AS is a crucial mechanism to “*regulate the regulators*”. Interestingly, we recently identified in circulating precursor cells new variants of *MED* genes involved in the formation of the Mediator complex, a key component of the transcription machinery [29]. Moreover, isoform switching, occurring during stem cell differentiation, has been demonstrated for key genes encoding transcriptional regulators, such as *OCT4*, *NANOG* and *FOXP1* [30,31,32,33]. Therefore, identifying and characterizing new variants of TFs genes is fundamental to better understand gene expression regulation in physiologic processes and/or pathologic states.

In this work, through a targeted re-analysis of our previously published RNA-Seq datasets [16] we identified and experimentally confirmed the presence of nine novel transcripts in seven TF-encoding genes. Using computational predictions we also characterized their putative protein products. Notably, all new TFs variants identified are generated by exon skipping events and all are predicted to encode novel protein isoforms. The transcription factors transcripts analyzed in this study had a wide expression pattern, not restricted to eEPCs where they have been identified. Nonetheless, we cannot exclude that they are differentially regulated—with respect to the canonical isoforms—nor that they have different structural and functional properties. Unfortunately, given the absence of structures in the PDB database and/or the fact that missing residues fall in non-functional domains, we could not predict protein structure and functionality for ARID5B, SP140L and NCOA2 proteins. In addition, although RNA-Seq data indicated two novel alternative transcripts of *IRF1* gene, we did not consider them for prediction analyses since they were already identified in a previous study.

Interestingly, our sequence-based predictions revealed that the alternative transcript of *SATB1* has a different translation start codon. SATB1 is a negative transcriptional regulator with the peculiar ability to integrate higher-order chromatin organization with the typical TF-mediated gene expression regulation. Indeed, it acts by binding to matrix attachment regions of DNA and inducing a local chromatin-loop remodeling. SATB1 expression positively correlates with tumor progression (reviewed in [34]). The increased activation of SATB1 determines the expression of genes involved in the epithelial-mesenchymal transition (EMT). Thus, as its aberrant expression promotes various types of cancers, the identification of a new SATB1 variant may be relevant for cancer-related studies. In particular, the new transcript is predicted to encode a shorter protein that lacks the first 72 aa and in turn the NLS. Thus, we predict that the new protein, unable to enter into the nucleus, may cause obvious effects on target gene expression. ELF2 (E74-Like Factor 2) is a member of the ETS gene family that is involved in the regulation of crucial B-, T-cell and vascular specific genes [35,36]. Moreover, ELF2, through interaction with AML1 (acute myeloid leukemia 1) protein, is able to trigger hematopoiesis and in turn is involved in the onset of leukemia. Cho and colleagues [37] used different ELF2 mutant proteins to understand ELF2 functions. Interestingly, one of these mutants (identical to our predicted new protein) was unable to transactivate the expression of an AML1 target gene. Thus, the novel ELF2 isoform described in our work (predicted to lack the transactivation domain) is likely to be a dominant negative variant of ELF2, acting to repress AML1-mediated transactivation. Therefore, this new ELF2 variant deserves further study to clarify its possible role in leukemia.

NCOA2 is a coactivator of nuclear hormone receptors involved in the onset of several diseases. Interestingly, different chromosomal aberrations affect its locus, carrying to the translation of chimeric proteins, such as PAX2-NCOA2 involved in rhabdosarcoma [38], HEY1-NCOA2 in chondrosarcoma [39], or the first identified MOZ-NCOA2 in acute myeloid leukemia [40]. The identification—and validation—of a new NCOA2 transcript is particularly relevant since loss of exons 12 and 13 causes a frameshift with PTC formation. Much evidence demonstrates that mRNAs in which PTC leads to the translation of a truncated protein undergo degradation through NMD machinery. Accordingly, the newly identified *NCOA2* transcript is a potential candidate to NMD-mediated degradation. On the contrary, if translated, the encoded protein would lack 665 aa at the *C*-terminus, determining the lack of dimerization ability mediated by two leucine-rich motifs. Thus, the novel NCOA2 isoform may act as dominant negative toward the canonical protein competing for binding to DNA.

Alternative splicing can also determine the acquisition of new domains and/or functions. Indeed, the most intriguing results of our study have been obtained for the *ZNF266* gene. Although we did not experimentally verify the presence of the new protein, different observations strengthen the hypothesis that it is effectively translated. The first evidence comes from our expression analysis. Indeed, the new alternative *ZNF266* transcript is expressed in all analyzed samples (cell lines and tissues) at high levels, comparable to—or even higher than—those of the canonical isoform (Supplementary Figure S4A). The 67 additional amino acids (at *N*-terminus) of the new predicted protein have high similarity scores with some paralogue ZNF genes and a high evolutionary conservation. Moreover, one of the most convincing pieces of evidence is that the presence of the new 67 aa determines the formation of the complete KRAB-A and KRAB-B box domains, that are encoded by different modular exons, as usually occurs. A similar situation—*i.e.*, the presence of proteins with a complete or truncated KRAB domain—is known for ZNF74 as well as other ZNFs [41]. Isoform switching of TFs variants is known to be a mechanism able to control gene expression. In this case, the new ZNF266 isoform (with complete KRAB-A and KRAB-B boxes) would act as a stronger transcriptional repressor compared to the annotated protein. Surface accessibility profile also suggested the presence of three additional C_2_H_2_ zinc finger motifs. All these findings indicate that the new *ZNF266* transcript is likely to be translated.

It is known that AS can modify not only the structural organization, but also protein functionality [12,13,27,28,29,30]. Therefore, our study highlights that AS gives a significant contribution to TF variant diversity. This could determine the loss—or gain—of specific protein functions, possibly in a time- and tissue-dependent manner. In this context, the rapid advance of sequencing techniques, that allow deep study of the transcriptome by RNA-Seq, is providing unprecedented advantages, even posing new challenges [42]. Notably, in this work we exclusively focused on exon skipping events, although we are aware that other physiological AS mechanisms may occur, as well as aberrant splicing and re-splicing events [43]. Of note, several reports have demonstrated that conserved alternatively spliced cassette exons are significantly shorter than constitutive ones [9,44], indicating that cassette exons are more prone to be alternatively spliced. However, most of the newly identified cassette exons in TF genes do not show this feature.

Additionally, we did not investigate the possibility that these new transcripts have new 3'UTRs, and we cannot exclude that they undergo differential regulation by miRNAs [45]. These new transcripts may also be differentially expressed in pathologic contexts, and it would be desirable to assess this hypothesis using innovative tools (e.g., AltAnalyze), as described by Soreq and colleagues [46].

In conclusion, in this study—merging computational analyses and experimental validations of RNA-Seq data—we identified and characterized new TF variants generated by alternative splicing. To strengthen their biological significance and clarify their effect on gene expression regulation, targeted biochemical studies are needed. Particularly, ChIP-Seq based studies could determine if the new TFs variants differ in their ability/specificity to bind target promoters. Addressing the potential role of the newly identified TF variants represents a crucial step towards understanding the regulatory mechanisms underlying physiological states as well as human diseases, especially cancer and metabolic disorders.

## 4. Experimental Section

### 4.1. Computational Analysis of RNA-Seq Datasets

A complete list of 1529 TF-encoding genes was retrieved from the TFClass database [18]. Uniquely Mapped Reads (UMRs) from our previous published datasets [16] were used to quantify the expression of TF-encoding genes and to identify novel TF transcripts. Cassette exon splicing was detected as described elsewhere [17]. Briefly, TF *de novo* identified splice junctions were intersected with exons annotation (RefSeq, UCSC, Ensembl and Gencode) and then classified in seven different categories. The most common mechanism of AS was the exon skipping. To be more confident, we set an arbitrary threshold of two reads mapping on the novel splice junction in at least one RNA-Seq dataset. UCSC Genome Browser [47] was used to visualize and select new putative splicing events for the validation. Matching of the new transcripts with human spliced and un-spliced ESTs, as well as with AceView gene predictions was considered as a selection parameter. Splice junctions overlapping repeats families were discarded.

### 4.2. Cell Cultures

HEK293, MCF7 and HeLa cell lines were cultured in Dulbecco’s Modified Eagle Medium (DMEM) supplemented with 10% fetal bovine serum (FBS), 2  mmol/L l-glutamine, 100 U/mL penicillin, and 100 μg/mL streptomycin (GIBCO/BRL Life Technologies, Grand Island, NY, USA). Cells were incubated at 37 °C in a 5% CO_2_ and humidified atmosphere.

### 4.3. RNA Extraction and RT-PCR Assays

Total RNA was isolated from HEK293, MCF7, and HeLa cell lines using a standard TRIZOL (Invitrogen, Carlsbad, CA, USA) protocol as previously described [16], whereas RNAs of eEPC, MCF10 cells and monocytes were already available from previous studies [16,23]. RNA integrity and concentration were evaluated by gel electrophoresis and spectrophotometry (NanoDrop Technologies, Rockland, DE, USA).

Reverse transcription was performed on 1 µg of total isolated RNA for each cell line with SuperScript II Reverse Transcriptase (Invitrogen), using Oligo(dT)_12–18_ primer in a 20 µL reaction, according to manufacturer’s protocol. cDNAs (1 µL) were used as template for PCR assays with isoform-specific primers, designed with Oligo 4.0 software (National Biosciences Inc., Plymouth, MN, USA, Table 1), in standard PCR reaction with 2.5 U AmpliTaq-Gold (Life Technologies, Gaithersburg, MD, USA; described in [48]).

### 4.4. DNA Gel Extraction, Cloning and Sequencing

DNA fragments obtained by PCR amplification were purified from agarose gel using centrifugal filter devise Ultrafree-DA (Millipore, Bedford, MA, USA), according to the manufacturer’s instructions. Where necessary, DNA amplicons were cloned into pCR II TOPO TA vector (Invitrogen). Purified DNA fragments and clones were directly sequenced by Sanger method on ABI PRISM^®^ 3730 DNA Analyzer sequencers (Applied Biosystems, Foster City, CA, USA), confirming the specificity of reactions.

### 4.5. In Silico Analysis

ApE [49] software was used to analyze and assemble ABI sequences trace files of the newly identified TF transcripts. Exonic structure and genomic position of the novel transcripts were reconstructed using the BLAT tool at UCSC Genome Browser [50].

ApE was used also to find putative ORFs in the new TFs transcripts and to predict the related protein product, whereas the amino acid sequences of the canonical TFs isoform were retrieved from the UniProt database [51]. Newly identified and annotated TFs sequences were aligned using ClustalW2 [52] with default parameters. Relative surface accessibility (RSA) of the ZNF266 new isoform and the corresponding *Z*-scores were predicted using NetSurfP server (version 1.1; RSA values in the Supplementary File S2) [53]. The Homology Modeling (HM) was carried out submitting TFs sequences to the Web server I-TASSER [54]. The lack of close homologues annotated structures as useful templates in this analysis, caused the low accuracy of the model. Obtained models were visualized and rendered using the PyMol system (Schrödinger LLC, Jersey City, NJ, USA).

## Supplementary Materials

Supplementary materials can be found at http://www.mdpi.com/1422-0067/16/01/1755/s1.
